# Supplementary material for: Investigating the use of a one-page infographic to improve recruitment and retention to the BASIL+ randomised controlled trial: A Study Within a Trial (SWAT)
Source: PLoS One. 2025 Sep 3;20(9):e0320821. doi: 10.1371/journal.pone.0320821 (PMC12407392; doi:10.1371/journal.pone.0320821)
Supplement: Attachment — Submitted filename: BASIL_ SWAT V2 Response to Comments.docx [file pone.0320821.s004.docx]

Dear Editor,

Thank you for your valuable feedback on our manuscript, Investigating the use of a one-page infographic to improve recruitment and retention to the BASIL+ randomised controlled trial: A Study Within a Trial (SWAT). We appreciate your time and thoughtful comment, which have helped us improve the quality of our paper.

We are pleased to submit a revised version of the manuscript that incorporates all suggested revisions. We have addressed each comment individually, as detailed below.

**Reviewer 2 Comment**

**Comment:** It is not normal practice to report an ICER when the intervention is dominated - suggest that you say something along the lines of...."The intervention was associated with higher costs (£821.52 per site) and a lower recruitment rate (3.6% vs 4.2%) than the control.
This indicates that the intervention was more costly and less effective, and therefore dominated by the control.
As a result, an ICER is not presented, as it would not provide meaningful decision-making information."

**Response**: We are grateful to Reviewer 2 for their constructive feedback. We agree that the inclusion of an ICER when the intervention is dominated adds little to the decision-making process. Despite this being part of our original outcome plan, we have removed the ICER as suggested and have reworded the text accordingly.

We believe the revised manuscript is significantly stronger thanks to the reviewers' feedback. Thank you again for your time and consideration. We look forward to hearing from you.

Sincerely,

Lucy Atha, on behalf of the BASIL+ Trial Team.
